# Supplementary material for: A New Light on Potential Therapeutic Targets for Colorectal Cancer Treatment
Source: Biomedicines. 2021 Oct 10;9(10):1438. doi: 10.3390/biomedicines9101438 (PMC8533612; doi:10.3390/biomedicines9101438)
Supplement: Supplementary file 1 [file biomedicines-09-01438-s001.zip › biomedicines-1357608-supplementary.pdf]

A New Light on Potential Therapeutic Targets for Colorectal Cancer Treatment

Wei-Lun Tsai [1,†], Chih-Yang Wang [2,3,†], Yu-Cheng Lee [4], Wan-Chun Tang [2,3], Gangga Anuraga[1,3,5], Hoang-Dang-Khoa-Ta [1,3], Yung-Fu Wu [6], Kuen-Haur Lee[2,3,7,*]

1 PhD Program for Cancer Molecular Biology and Drug Discovery, College of Medical Science and Technology, Taipei Medical University and Academia Sinica, Taipei 11031, Taiwan; mebar1995@gmail.com (W.L.T.); g.anuraga@unipasby.ac.id (G.A.); d621109004@tmu.edu.tw (H.-D.-K.T.)
2 PhD Program for Cancer Molecular Biology and Drug Discovery, College of Medical Science and Technology, Taipei Medical University, Taipei 11031, Taiwan; chihyang@tmu.edu.tw (C.-Y.W.); yeas0310@hotmail.com(W.-C.T.)
3 Graduate Institute of Cancer Biology and Drug Discovery, College of Medical Science and Technology, Taipei Medical University, Taipei 11031, Taiwan
4 Graduate Institute of Medical Sciences, College of Medicine, Taipei Medical University, Taipei 11031, Taiwan; yclee0212@tmu.edu.tw
5 Department of Statistics, Faculty of Science and Technology, Universitas PGRI Adi Buana, Surabaya, East Java 60234, Indonesia
6 Department of Medical Research, Tri-Service General Hospital, School of Medicine, National Defense Medical Center, Taipei 11490, Taiwan; qrince@yahoo.com.tw
7 Cancer Center, Wan Fang Hospital, Taipei Medical University, Taipei 11031, Taiwan
* Correspondence: khlee@tmu.edu.tw
† These authors contributed equally to this work.

Supplemental Information includes two Supplemental Tables and one Supplemental Figure

Supplementary Table S1. Sequences of small interfering (si)RNAs and primers

**Sequences of siRNAs**		
**Gene name**	**Sense strand (5′-3′)**	**Antisense strand (5′-3′)**
CRNDE 1#	GGGUAUUCCUGUUUAUAGAT T	UCUAUAAACAGGAAUACCCAA
CRNDE 2#	GUCUGCAAUUCAUAAUGGAT T	UCCAUUAUGAAUUGCAGACTT

**Sequence of primers**		
**Gene name**	**Forward (5′-3′)**	**Reverse (5′-3′)**
CRNDE	ATATTCAGCCGTTGGTCTTTGA	TCTGCGTGACAACTGAGGATTT
MYLK	GTCTTATGTTATCTTCCATTC TA	TATAATAAACTGTGGCAATAC TG
GPX3	GCCGGGGACAAGAGAAGT	GAGGACGTATTTGCCAGCAT
ANGPTL4	TCTCTGGAGGCTGGTGGTTT	ACGCCTCTAGAGTCTGAGCA

Supplementary Table S2. Top 20 upregulated genes in colorectal carcinoma (CRC) (GSE21815)

T/N ratio	Gene symbol	Gene name
108.65	*DPEP1*	dipeptidase 1 (renal)
67.72	*KRT80*	keratin 80
59.75	*FABP6*	fatty acid-binding protein 6, ileal
56.90	*NKD2*	naked cuticle homolog 2 (*Drosophila*)
56.08	*FOXQ1*	forkhead box Q1
51.98	*KIAA1199*	KIAA1199 (CEMIP)
41.53	*ETV4*	ets variant 4
31.95	*TESC*	tescalcin
30.61	*FUT1*	fucosyltransferase 1 (galactoside 2-alpha-L-fucosyltransferase, H blood group)
30.49	*GAS2*	growth arrest-specific 2
29.49	*LOC100287482*	uncharacterized protein LOC100287482
29.03	*CRNDE*	colorectal neoplasia differentially expressed (non-protein coding)
28.73	*EPHX4*	epoxide hydrolase 4
26.53	*LY6E*	lymphocyte antigen 6 complex, locus E
25.31	*C2*	complement component 2
24.96	*TRIB3*	tribbles homolog 3 (*Drosophila*)
24.33	*TGFBI*	transforming growth factor, beta-induced, 68 kDa
20.96	*TDGF1*	teratocarcinoma-derived growth factor 1
19.64	*AJUBA*	ajuba LIM protein
18.95	*TUBB3*	tubulin, beta 3 class III

T/N, tumor/normal.

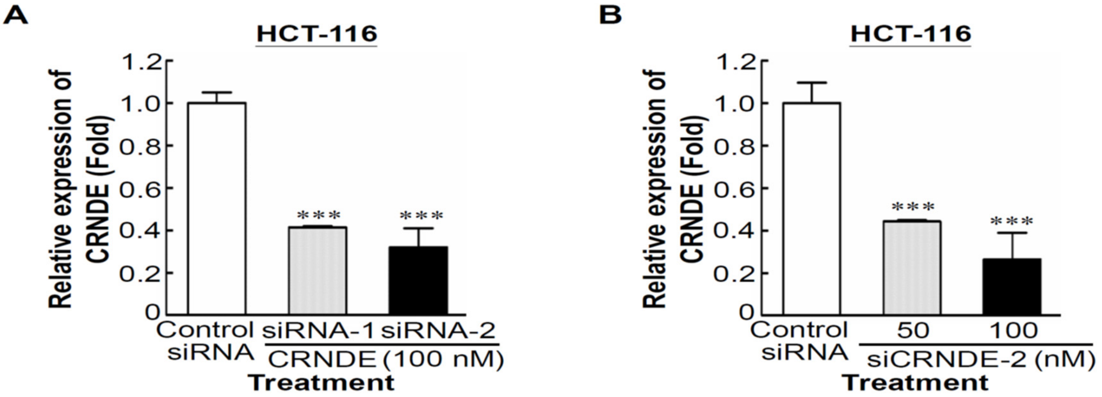

Supplementary Figure S1. The knockdown efficiency of CRNDE. (**A**) The knockdown efficiency of CRNDE by CRNDE siRNA #1 and #2. (**B**) The knockdown efficiency of CRNDE by CRNDE siRNA #2 at the concentration of 50 or 100 nM.
